# A Cystic Surprise: Unearthing Fimbrial Cysts as an Uncommon Source of Abdominal Pain

**DOI:** 10.7759/cureus.49885

**Published:** 2023-12-04

**Authors:** Srishti Choudhary, Varsha Kose

**Affiliations:** 1 Department of Obstetrics and Gynaecology, NKP Salve Institute of Medical Sciences and Research Centre and Lata Mangeshkar Hospital, Nagpur, IND

**Keywords:** exploratory laparotomy, hydrosalpinx, adnexal pathologies, paraovarian cyst, fimbrial cyst

## Abstract

Fimbrial cysts also known as paraovarian cysts are small and asymptomatic and are occasionally large resulting in pelvic pain. It is difficult to differentiate a fimbrial cyst from an ovarian cyst by imaging; therefore, they are often recognized intra-operatively during laparotomy. This report presents a rare case of a 48-year-old female who presented with the primary complaints of persistent right lower abdominal pain that was intermittently radiating to the back for one year. Clinical findings reported the possibility of twisted right hydrosalpinx but the tumor biomarkers were found to be within the normal range. In addition to this, ultrasound sonography (USG) and magnetic resonance imaging (MRI) revealed a pelvic mass that was indicative of cystic lesions. As the above-mentioned diagnosis was found to be challenging, exploratory laparotomy as a part of surgical intervention and diagnosis was performed along with histopathological investigations that confirmed the existence of fimbrial end cysts on both sides. Fimbrial end cysts represent a rare yet significant cause of abdominal pain; therefore, early recognition, thorough clinical evaluation, and appropriate diagnostic workup are essential for timely intervention and preventing potential complications associated with fimbrial end cysts.

## Introduction

Fimbrial cysts also known as paraovarian cysts (POCs) consist of 10% of adnexal masses approximately [1]. These are more prevalent among women in 30-40 years of age and are usually small and asymptomatic leading to pelvic pain [2]. Fimbrial cysts are unilocular, thin-walled, and typically originate in the broad ligament. Whereas, ovarian serous cystadenomas can have similarities with multilocular cysts [3]. Because it can be challenging to accurately distinguish between an ovarian cyst and a fimbrial cyst using imaging, these cysts are frequently found intraoperatively during laparotomy.

A paratubal cyst is a closed sac filled with fluid that develops next to the fallopian tube and ovary, but it is never connected to them [4]. It is often unilateral and benign, and it is situated at the ligament that connects the uterus and the ovary [5]. POCs are classified as either of mesonephric origin, paramesonephric, or mesothelial (peritoneal inclusions) based on histopathology testing [2]. However, using imaging techniques to diagnose POCs preoperatively has frequently proven challenging as they are often misdiagnosed as ovarian cysts as the neoplastic POCs originate from a neoplastic transformation of a paraovarian simple cyst or the adjacent ovary. Whereas, diagnosing adnexal masses or neoplasms with magnetic resonance imaging (MRI) has proven beneficial. Increased knowledge of the MRI features associated with POCs could result in MRI playing a significant role in avoiding surgery by offering a noninvasive diagnosis [2]. Hence, this report highlights a rare case of fimbrial cyst along with its diagnostic procedures and management.

## Case presentation

A 48-year-old female presented with the primary complaints of persistent right lower abdominal pain that intermittently radiated to the back for one year. She stated perplexing concomitant symptoms of sporadic spotting for the past four months despite the postmenopausal status that extended for 1.5 years. The obstetric history consisted of two live births, three abortions, and tubal ligation.

On abdominal examination, localized tenderness in the right lower abdomen and a small suprapubic mass on the right side was observed. Pelvic and vaginal examination revealed tenderness in the right fornix. Based on the above findings, a clinical diagnosis of a twisted right hydrosalpinx was reported. Additionally, investigations related to tumor biomarkers consisting of alpha-fetoprotein (5.73 ng/ml), serum prolactin (14.4 ng/ml), lactate dehydrogenase (168 IU/L), and CA-125 (7.28) were all found to be within the normal range.

**Figure 1:**
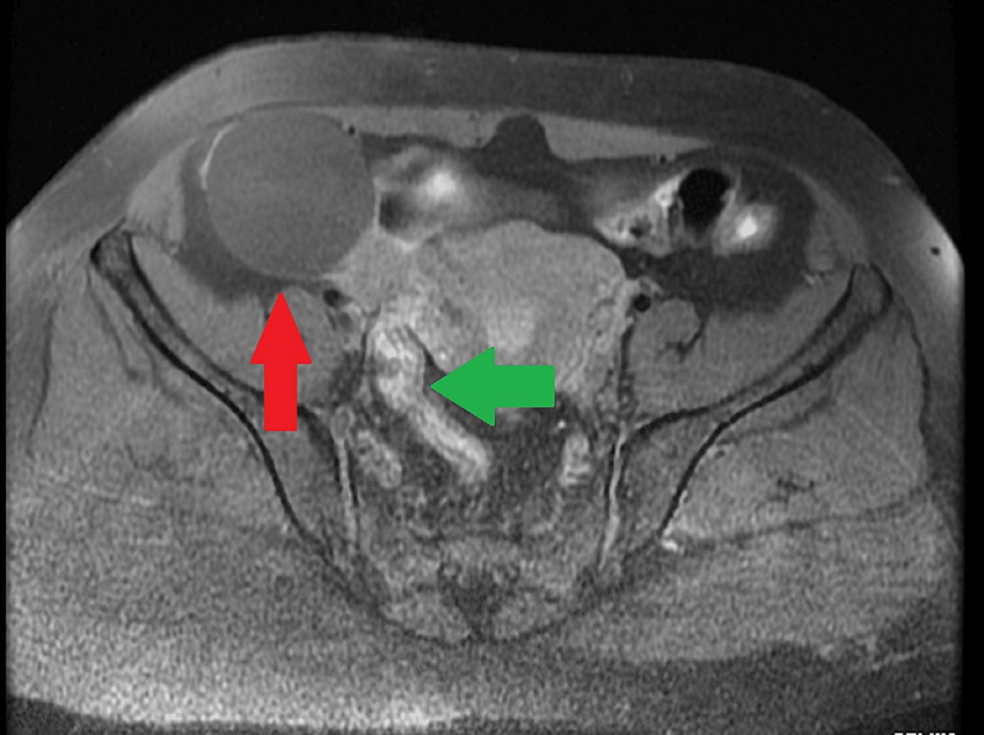
Magnetic resonance imaging (MRI) illustrating fimbrial cyst. Red arrow = Right side ovarian cyst of size 5.2 cm × 4.8 cm × 5.3 cm; Green arrow = Right side fallopian tube with a cystic lesion of width 1.4 cm.

Furthermore, ultrasonography (USG) was performed that illustrated an anechoic cystic lesion which measured 4.8 cm in the right adnexa, that raised a suspicion of either a right ovarian cyst or hydrosalpinx. Subsequently, to confirm the diagnosis, an MRI was performed that demonstrated the bilateral presence of hydrosalpinx and a right-sided simple ovarian cyst of size 5.2 cm × 4.8 cm × 5.3 cm as illustrated in Figure 1.

Endocervical curettage and cervical biopsy were performed in light of the patient's postmenopausal spotting, uncovering a proliferative endometrial pattern. Though offered medical management, the patient declined such intervention, thus necessitating exploratory laparotomy.

**Figure 2:**
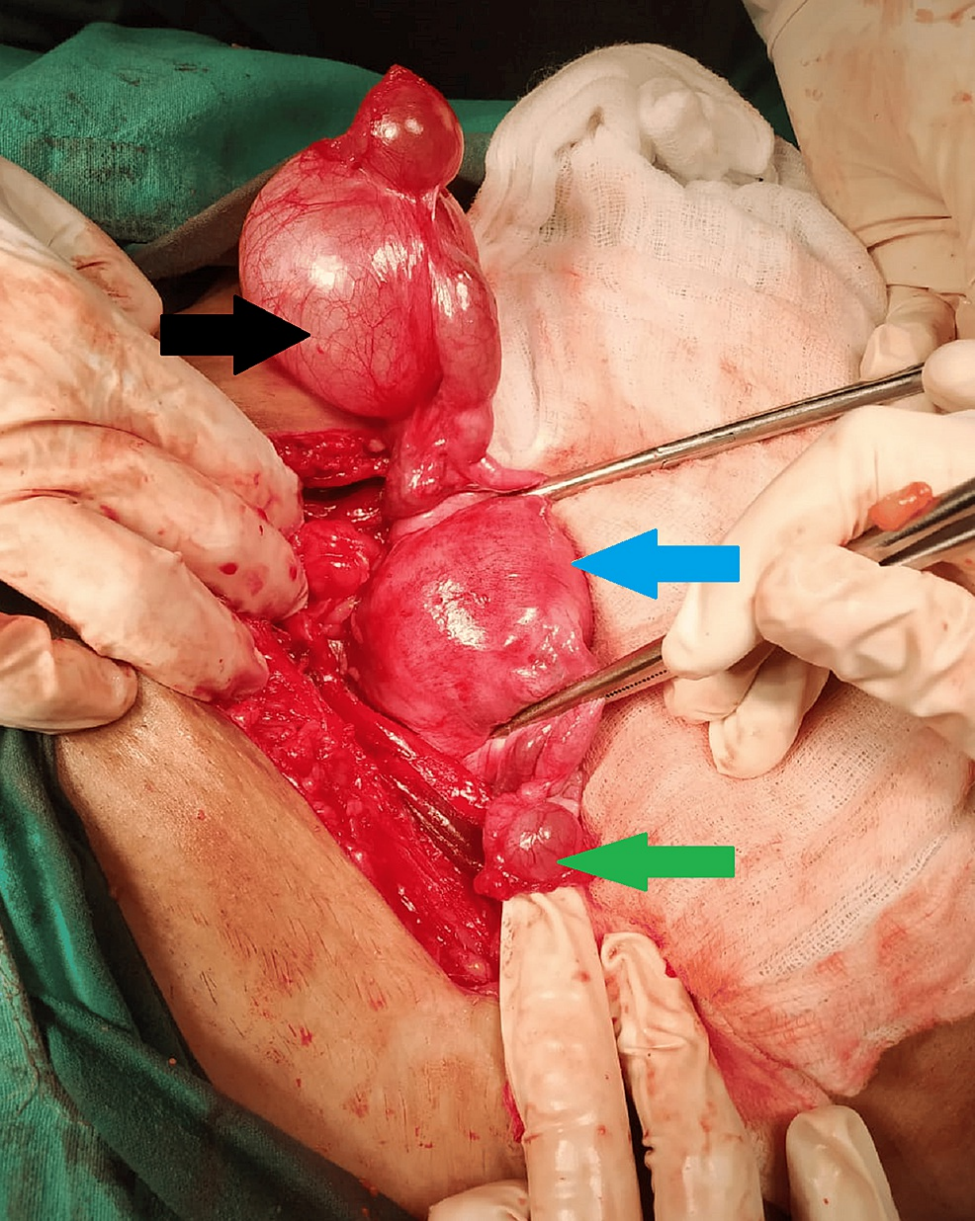
Intraoperative findings illustrating fimbrial cyst. Blue arrow = Uterus between the two forceps; Black arrow = Fimbrial end cyst with cystic swelling on the right side of 5.5 cm × 5 cm in size; Green arrow = Fimbrial end cyst with cystic swelling on the left side of 3 cm × 3 cm in size.

Before commencing exploratory laparotomy the procedure was explained to the patient and written informed consent was obtained. Remarkably, intraoperative findings demonstrated the existence of fimbrial end cysts on both sides, with measurements of 5.5x5 cm on the right and 3x3 cm on the left as shown in Figure 2. The histopathology report confirmed the presence of fimbrial end cysts on both sides. The patient was resistant to follow-up because of the lack of awareness regarding the disease therefore as a precautionary measure a total abdominal hysterectomy, bilateral salpingectomy, and left oophorectomy were performed. The diagnosis performed for the present case was found to be challenging as the tumor biomarkers were within the normal range but the imaging studies reported the presence of cystic lesions. Therefore, to confirm the diagnosis exploratory laparotomy as a part of surgical intervention and diagnosis was performed along with histopathological investigations that confirmed the existence of fimbrial end cysts on both sides.

## Discussion

In the present case, the patient reported symptoms of abdominal pain with localized tenderness for which clinical examination reported the possibility of twisted right hydrosalpinx. The diagnosis performed for the present case was found to be challenging as the tumor biomarkers were within the normal range but the imaging studies reported the presence of cystic lesions. Therefore, to confirm the diagnosis exploratory laparotomy as a part of surgical intervention and diagnosis was performed along with histopathological investigations that confirmed the existence of fimbrial end cysts on both sides. As stated by a previous study, POCs may occur in patients who have bilateral tubal ligation [4] which was found to be congruous with the present case, as the obstetric history reported by the patient described tubal ligation which can be considered as a cause of POCs. Therefore, while determining the differential diagnosis of acute abdomen in women, POCs must be considered [4].

The majority of patients report no symptoms but the most common symptoms include lower abdominal pain and discomfort, menstrual irregularities, abdominal distension, and the pressure effects of a large cyst, torsion, or rupture [6]. Previously reported studies demonstrated that postmenopausal bleeding, dysmenorrhea or irregular menstruation, and intermittent lower abdominal pain have all been linked to fallopian tube teratomas [7]. Whereas, in the present case, the patient reported symptoms of persistent right lower abdominal pain that intermittently radiated to the back for one year. In addition to this, she stated perplexing concomitant symptoms of sporadic spotting for the past four months despite the postmenopausal status that extended for 1.5 years. Hence, the above-mentioned symptoms should be taken into consideration while performing a clinical examination.

POCs can display a variety of sonographic characteristics. Sonographically, they are often unilocular cysts with thin walls and smooth margins. If transvaginal sonography reveals no papillary projections, their risk of malignancy is minimal. However, pathological testing may reveal a borderline tumor if mural proliferations are present. Whereas, for preoperative diagnosis, MRI can be useful [8] but only one out of every fifteen patients has a suspected fimbrial cyst before surgery [1]. Whereas, in the present case, both USG and MRI were performed in which USG disclosed an anechoic cystic lesion which measured 4.8 cm in the right adnexa, that raised a suspicion of either a right ovarian cyst or hydrosalpinx. Subsequently, MRI findings reported the bilateral presence of hydrosalpinx and a right-sided simple ovarian cyst of size 5.2 cm × 4.8 cm × 5.3 cm. Therefore, to confirm the diagnosis exploratory laparotomy was performed along with the histopathological investigation that confirmed the existence of fimbrial end cysts or POCs on both sides.

Torsion (2-16%), hemorrhage, rupture, and secondary infection are among the complications associated with POCs. Neoplastic transformation (2.9%) includes endometriod cystadenocarcinoma, mucinous cystadenocarcinoma, papillary serous cystadenoma, and serous cystadenocarcinoma [9]. Another case demonstrated a twisted fimbrial cyst in a 22-year-old female for which exploratory laparotomy was performed and a histological examination was performed to confirm the diagnosis [10]. Similarly in the present case, exploratory laparotomy along with histopathological analysis confirmed the diagnosis of sizeable fimbrial end cysts on both sides.

Fimbrial end cysts often present clinical and radiological features that can be misleading, leading to their misclassification as other conditions, such as hydrosalpinx or ovarian cysts. While conservative management involving medical treatment may be contemplated for certain patients, surgical intervention often emerges as the preferred approach, particularly in the case of symptomatic cysts. Surgical options consisting of laparoscopy or laparotomy [11,12], with the choice influenced by the extent and location of the cysts, are preferred and reported excellent results as the patient adhered well to the management. Similarly in the present case, endocervical curettage and cervical biopsy were performed in light of the patient's postmenopausal spotting, which uncovered a proliferative endometrial pattern. Though offered medical management, the patient declined such intervention, thus necessitating exploratory laparotomy which was performed on the patient that confirmed the presence of a fimbrial cyst. Hence, it is essential to recognize that the presentation of fimbrial end cysts lacks specificity and may overlap with symptoms of other gynecological or abdominal conditions, thus contributing to diagnostic complexity as accurate diagnosis of fimbrial end cysts is essential for ensuring appropriate management.

## Conclusions

The present case was reported of a 48-year-old female who presented with the chief complaints of abdominal pain along with concomitant symptoms of sporadic spotting for the past four months despite her postmenopausal status. On investigations, and during intraoperative findings, the existence of fimbrial end cysts on both sides was confirmed. Fimbrial end cysts represent a rare yet significant cause of abdominal pain. Hence, early recognition, thorough clinical evaluation, and appropriate diagnostic workup are essential for timely intervention and preventing potential complications associated with fimbrial end cysts to ensure optimal patient care.
